# Chidamide Accelerates the Death of Senescence‐Like Diffuse Large B‐Cell Lymphoma Cells With TP53 Mutation Induced by Doxorubicin

**DOI:** 10.1096/fj.202500962RR

**Published:** 2025-10-22

**Authors:** Yun‐yan Yin, Yu Liu, Yi‐qi Lu, Zhi‐meng Tang, Zheng‐xin Zhu, Meng‐yuan Zhu, Hong‐yu Chen, Hui Hui, Jing‐yan Xu, Hui Li

**Affiliations:** ^1^ Department of Hematology China Pharmaceutical University Nanjing Drum Tower Hospital Nanjing People's Republic of China; ^2^ State Key Laboratory of Natural Medicines, Jiangsu Key Laboratory of Carcinogenesis and Intervention China Pharmaceutical University Nanjing People's Republic of China

**Keywords:** chidamide, DLBCL, doxorubicin, P53 mutation, senescence

## Abstract

Diffuse large B‐cell lymphoma (DLBCL), a heterogeneous malignancy characterized by distinct genetic and clinical subtypes, remains a therapeutic challenge despite standard R‐CHOP chemotherapy. This study systematically investigated the synergistic anti‐tumor efficacy and mechanism of the histone deacetylase inhibitor chidamide (CHI) combined with doxorubicin (DOX) in DLBCL models. Through multimodal experimental approaches including CCK8 proliferation assays, senescence‐associated β‐galactosidase (SA‐β‐gal) staining analysis, comet assay for DNA damage quantification, and comprehensive western blotting for molecular mechanism elucidation, we identified a potent synergistic interaction between CHI and DOX. By constructing a xenograft tumor model, the synergistic effect of the two drugs and its mechanism in vivo were confirmed. Our research found that CHI showed cell cycle inhibition and apoptosis induction effects on DLBCL cells. When combined with DOX, both synergistically inhibited the proliferation of DLBCL cells and promoted death of DOX‐induced senescent cells. Preliminary mechanism studies revealed that CHI can reduce the level of mutant p53, inhibit cell senescence, and induce irreparable DNA damage in DOX‐induced senescent tumor cells, ultimately leading them into programmed apoptosis. Thus, the “One‐two punch” strategy increased the sensitivity of p53‐mutated DLBCL cells to CHI. In summary, our study demonstrates that CHI synergizes with DOX to suppress DLBCL and presents a promising strategy to augment the therapeutic efficacy of DOX.

## Graphical abstract

Combined Doxorubicin (DOX, senescence inducer) and Chidamide (CHI, HDAC inhibitor) treatment inhibits mutp53 function and synergistically eliminates tumor cells by suppressing mutp53, blocking senescence escape, and triggering irreparable DNA damage, leading to programmed death—a “one‐two punch” strategy sensitizing mutp53‐DLBCL to therapy.

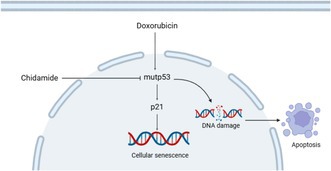

## 1 Introduction

Diffuse Large B‐cell lymphoma (DLBCL) is a highly heterogeneous and aggressive malignant tumor, accounting for 30%–40% of adult non‐Hodgkin lymphomas [1], making it the most common type of non‐Hodgkin lymphoma. The standard treatment for newly diagnosed DLBCL patients is R‐CHOP (rituximab, cyclophosphamide, doxorubicin, vincristine, prednisone). Although about two‐thirds of patients treated with first‐line regimens can be cured, one‐third still experiences relapse or metastasis due to drug resistance [2]. In DLBCL patients, approximately 30%–50% carry *TP53* gene mutations, including *TP53* mutations and/or del (17p), and these patients have a poor prognosis [3]. For DLBCL patients with *TP53* gene mutations, since standard R‐CHOP treatment cannot overcome the adverse prognosis of *TP53* mutations, finding more effective treatment options has become a key focus of current research to improve the prognosis of this group of patients [4]. Since research on drugs directly targeting *TP53* gene abnormalities has not yet entered clinical practice, many clinical studies focus on exploring strategies to utilize existing new drugs in combination with different drug regimens to improve the prognosis of patients with *TP53*‐mutated DLBCL. The “R‐CHOP+X” regimen is one of the important areas of exploration [5].

Doxorubicin (DOX) is a crucial cytotoxic agent in the R‐CHOP regimen, exerting its antitumor effect by inhibiting topoisomerase II and inducing DNA double‐strand breaks [6]. Following DNA damage caused by DOX, cancer cells rapidly activate the DNA damage response (DDR) [7]. The resulting DNA damage triggers cellular repair mechanisms, and inadequate repair can lead to apoptosis or senescence [8]. DOX is often limited in clinical practice due to cardiotoxicity. Low doses of DOX reduce its cytotoxic effect on tumor cells and more easily induce cells into a senescent state, increasing the risk of DLBCL recurrence [9]. Cellular senescence has been considered a beneficial cellular stress response in antitumor therapy, allowing cells to enter a stable growth arrest state when exposed to harmful stimuli, thereby preventing tumor cell proliferation and growth [10]. However, increasing evidence suggests that various therapies can induce different senescent cancer cells which may regain their proliferative capacity in vitro, potentially explaining tumor recurrence following treatment remission [11]. Therefore, selectively eliminating senescent tumor cells induced by therapy is a method to reduce tumor recurrence, known as senolytic therapy [12].

Chidamide (CHI) is an HDAC inhibitor used to treat relapsed and refractory peripheral T‐cell lymphoma, but it has not been approved for the treatment of DLBCL [13]. Studies have found that CHI exerts an anti‐DLBCL effect by inducing apoptosis [14], and combined chemotherapy can improve the overall survival rate of patients with DLBCL and significantly improve the clinical prognosis of non‐GCB DLBCL [15]. In addition, studies have reported that CHI combined with chemotherapeutic drugs can reduce the expression level of p53 protein so that a patient with DLBCL relapse with a *TP53* gene mutation can achieve sustained remission [16]. Therefore, it is speculated that the combination of CHI and low‐dose DOX with a senescence‐inducing effect may have the effect of lysing senescent cells and synergistically resisting DLBCL.

In the present study, we observed that CHI was able to inhibit DLBCL cell proliferation and induce apoptosis in p53‐mutated DLBCL cells. In addition, we also found that CHI can inhibit DOX‐induced cell senescence by reducing p53 protein levels, and the two drugs have strong synergistic effects in p53 mutant cell lines. These results provide a new scheme for improving the clinical treatment of p53 mutant DLBCL.

## 2 Materials & Methods

### 2.1 Cell Cultures

The human diffuse large B‐cell lymphoma cell lines RI‐1, SU‐DHL‐8, and OCI‐LY3 were purchased from the Cell Bank of Shanghai Institute of Biochemistry & Cell Biology. All cells were expanded and stored in liquid nitrogen upon receipt, and each aliquot was passaged fewer than 25–30 times in our laboratory. RI‐1 and SU‐DHL‐8 cells were cultured in RPMI‐1640 medium (Gibco, Invitrogen Corporation, Carlsbad, CA) supplemented with 10% FBS (Gibco), while OCI‐LY3 cells were cultured in IMDM medium (Gibco, Invitrogen Corporation, Carlsbad, CA) supplemented with 20% FBS (Gibco) in a humidified environment (Thermo Fisher Scientific, Waltham, MA) with 5% CO_2_ at 37°C. All experiments were performed with mycoplasma‐free cells.

### 2.2 Compounds and Reagents

CHI was purchased from Chipscreen Bioscience Company. DOX was purchased from Yunmei Technology Company. Both powders were dissolved in dimethyl sulfoxide (DMSO) to a concentration of 10 mM and stored at −80°C. The solution was diluted with basal medium to designated concentrations, and the final concentration of DMSO will not exceed 0.1%. Cells treated with the highest concentration of DMSO served as controls in corresponding experiments. Primary antibodies against β‐actin, PARP1, PUMA, and NOXA were obtained from Santa Cruz Biotechnology (Santa Cruz, CA). Primary antibodies against p53, p21, Mcl‐1, p‐p53 (S15), caspase‐3, cleaved caspase‐3, IL‐6, MDM2, Bxl‐XL, Bcl‐2, Bax, and γ‐H2AX were obtained from ABclonal Technology (Wuhan, China). Primary antibodies against Cyclin B, Cyclin D, and CDK2 were obtained from Proteintech (USA). The secondary antibodies HRP Goat Anti‐Rabbit IgG(H + L) and HRP Goat Anti‐Mouse IgG(H + L) were obtained from ABclonal Technology (Wuhan, China).

### 2.3 Cell Apoptosis Assays

The cells were collected and labeled with Annexin V and propidium iodide (PI) according to the protocols of Annexin V/PI Cell Apoptosis Detection Kit (Vazyme Biotec, Nanjing, China). The cells were resuspended in binding buffer (50 μL), added 2 μL of annexin V‐FITC and PI, and incubated in the dark for 15 min. The number of apoptotic cells was then measured by FACS Calibur flow cytometry (Becton‐Dickinson), and the data analysis was performed by FlowJo software (Tree Star, USA).

### 2.4 Cell Viability Assay

Cell viability was assayed in triplicate using the Cell Counting Kit‐8 (Vazyme, Nanjing, China). Cells were seeded in 96‐well plates and treated with CHI and/or DOX. Then, 10 μL of CCK8 solution was added to each well and incubated for 1–4 h at 37°C. Each group consisted of six parallel wells. Absorbance was measured at 450 nm using a SynergyTM HT multi‐mode reader (Bio‐Tek, Winoosky, VT). IC_50_ values were defined as the concentration that caused 50% inhibition of cell viability and were calculated using GraphPad Software.

### 2.5 Cell Cycle Assay

The cells were harvested and fixed in 70% pre‐cooled ethanol at 4°C. After fixation, the cells were washed with PBS buffer and stained with 20 μg/mL RNase A for 15 min at 37°C, followed by staining with 20 μg/mL PI in the dark for 30 min. The cells were then quantified using a FACS Calibur flow cytometer (Becton‐Dickinson). The distribution of cells in the S, G0–G1, and G2–M phases of the cell cycle was analyzed using FlowJo software (Tree Star, USA).

### 2.6 Senescence‐Associated β‐Galactosidase (SA‐β‐Gal) Assay

SA‐β‐Gal activity was measured using a β‐Galactosidase Staining Kit (Yeasen, Shanghai, China) according to the standard protocol. Cells were treated with CHI and/or DOX, washed once with phosphate‐buffered saline (PBS), fixed for 15 min, and incubated overnight at 37°C in the staining solution. The next day, the cells were washed twice with PBS and left to dry. The stained cells were observed and photographed under a microscope (Carl Zeiss, Germany).

### 2.7 Synergy Assay

The therapeutic effect of CHI and DOX combination was assessed using cell viability assays. CompuSyn software was used for the Chou‐Talalay analysis to evaluate the synergistic effects. Combination index (CI) values were used to indicate the effects of combination treatments and were used to resolve synergistic (CI < 0.9), additive (0.9 < CI < 1.1), and antagonistic (CI > 1) effects.

### 2.8 Quantitative Real‐Time PCR (qRT‐PCR)

RT‐qPCR was performed according to the manufacturer's instructions.

The primer sequences (5′‐3′) were listed below:

*GAPDH*.

Forward 5′‐GCAGGGGGGAGCCAAAAGGG‐3′.

Reverse 5′‐TGCCAGCCCCAGCGTCAAAG‐3′.

*TP53*.

Forward 5′‐GAGGTTGGCTCTGACTGTACC‐3′.

Reverse 5′‐TCCGTCCCAGTAGATTACCAC‐3′.

### 2.9 Western Blotting

Cells were collected and lysed in RIPA buffer (Thermo Scientific, USA) containing 1% protease/phosphatase inhibitors (GLPBIO, USA) on ice for 40 min. The tube was flicked every 20 min to ensure complete lysis. The lysed cells were then clarified by centrifugation at 12 000 rpm (5430R; Eppendorf, Hamburg, Germany) for 20 min at 4°C. The protein concentration in the supernatant was measured using the BCA Protein Assay Kit and a Varioskan multimode microplate spectrophotometer (Thermo Waltham, USA). Then equal amounts of protein extracts (20 μg) were separated by 8%–12% SDS‐polyacrylamide gel electrophoresis (SDS‐PAGE) and transferred to nitrocellulose membranes (NC) (Millipore, Boston, MA, USA). The membranes were blocked with 3% BSA in PBS at room temperature for 1 h and incubated overnight at 4°C with primary antibodies. The membranes were then incubated with an HRP goat anti‐rabbit IgG(H + L) or HRP goat anti‐mouse IgG(H + L) secondary antibody at room temperature for 1 h. Chemiluminescence detection was performed using ECL reagents (Thermo Fisher Scientific, USA) and imaged using the Amersham Imager 600 (GE, Piscataway, NJ, USA).

### 2.10 Comet Assay

Preheated 30 μL of 1% normal melting point agarose was spread on adhesive glass slides and set at 4°C for 10 min as a matrix. Cells were collected and diluted to 1 × 10^6^/mL and 10 μL of cell suspension was mixed with 75 μL of 0.7% low melting point agarose at 37°C, subsequently added 70 μL dropwise to the first layer of gel, and left at 4°C for 10 min as the second layer. When the second layer solidified, 0.7% low melting point agarose preheated at 37°C with 75 μL was added and placed at 4°C for 10 min as the third layer. The glass slides containing samples were immersed in the pre‐cold lysis solution at 4°C for an hour. After that, slides were placed in a horizontal electrophoresis tank filled with pre‐cold electrophoresis buffer and incubated for 20 min in the dark for DNA melting, followed by electrophoresis (25 V, 30 min). The slides were washed with neutralization buffer for 5 min × 3 times and stained with GelRed for 20 min in the dark and subsequently observed and snapped under the microscope (Carl Zeiss, Germany). Comet trails were analyzed by ImageJ plugins opencomet. Comet tail length was used to measure the DNA damage degree.

### 2.11 Human DLBCL Xenograft Mouse Model

The female BALB/c nude mice (4 weeks old, weighing 16–20 g) were supplied by Cavens Changzhou Laboratory Animal Center (Jiangsu, China). SU‐DHL‐8 cells (2.5 × 10^8^) were injected into nude mice through tail vein, and four nude mice were randomly selected to be injected with 0.9% normal saline intravenously as a blank group. After 2 days, mice engrafted with SU‐DHL‐8 cells were divided randomly into four groups (*n* = 4 per group): a control group, a CHI‐treated group, a DOX‐treated group, and a combination group. The mice in CHI groups were administrated CHI orally (25 mg/kg, every day for 2 weeks). The mice in DOX‐treated group were intraperitoneally injected with DOX (0.2 mg/kg, every 3 days for 2 weeks). The body weight of mice was measured every 2 days during the dosing period. Finally, the animals were sacrificed, and the spleens were prepared for flow cytometric analysis after the cells labeled with human CD45‐PE (huCD45) The staining of huCD45 is used to determine the infiltration of SU‐DHL‐8 cells. The animal study was carried out according to the regulations of the China Food and Drug Administration (CFDA) on Animal Care.

### 2.12 Statistical Analysis

All data were expressed as mean ± standard error of the mean (SEM) and obtained from three independent experiments performed in a parallel manner. Statistical analysis of multiple group comparisons was performed by one‐way analysis of variance (ANOVA) followed by the Tukey's multiple comparisons test. Comparisons between the two groups were analyzed using two‐tailed Student's *t*‐tests. The *P* values were indicated on the graph and *p* < 0.05 was considered statistically significant.

## 3 Results

### 3.1 The Inhibitory Effect of CHI on DLBCL Cells

**FIGURE 1:**
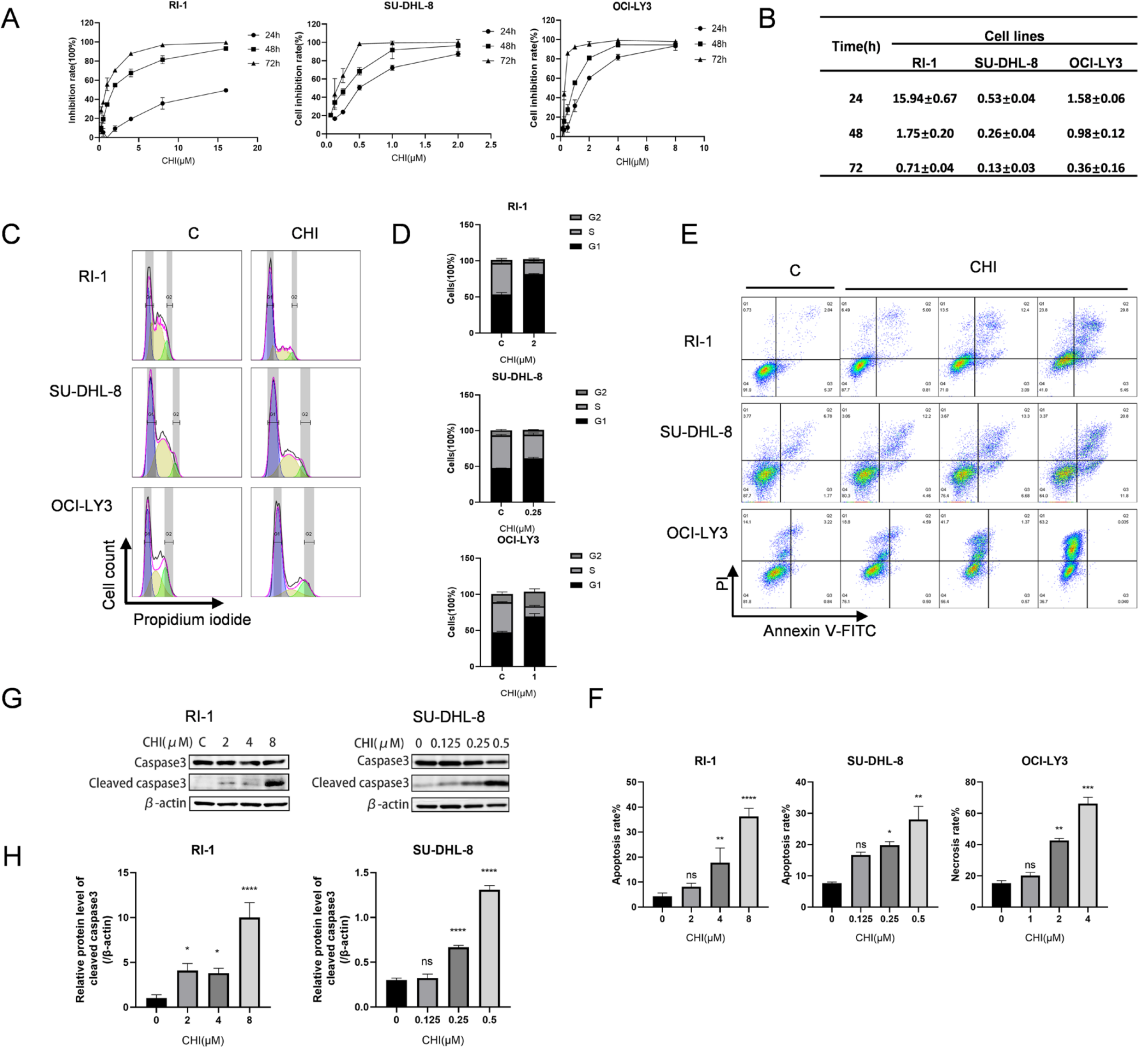
The inhibitory effect of CHI on DLBCL cells. (A) RI‐1, SU‐DHL‐8, and OCI‐LY3 cells were treated with different concentrations of CHI for 24 h, 48 h, and 72 h. (B) The IC50 values (μM) were calculated by GraphPad Prism 9.0. Data represent the mean ± SEM from three independent experiments. (C) RI‐1, SU‐DHL‐8, and OCI‐LY3 cells were treated with 2 μM, 0.25 μM, and 1 μM CHI for 48 h, respectively. DNA was stained by PI, and the DNA content was measured by flow cytometry. (D) Quantitative analysis of the proportion of cells in each cycle. Data represent the mean ± SEM from three independent experiments. (E) RI‐1, SU‐DHL‐8, and OCI‐LY3 cells were treated with different concentrations of CHI for 48 h; then, Annexin V‐FITC/PI staining was determined by flow cytometry. (F) Quantitative analysis of apoptotic cells. Columns represent the mean from three parallel experiments (mean ± SEM, *n* = 3). ns *p* > 0.05, *p < 0.05, **p < 0.01, ***p < 0.001. (G) RI‐1 and SU‐DHL‐8 cells were treated with different concentrations of CHI for 48 h. The expression of caspase‐3 and its active form were detected by western blot. (H) Relative protein expression level of cleaved caspase‐3. Columns represent the mean from three parallel experiments (mean ± SEM, *n* = 3). ns *p* > 0.05, *p < 0.05, **p < 0.01, ***p < 0.001.

To evaluate the growth inhibitory effects of CHI on DLBCL cell lines, we first examined cell viability by CCK8 assay. The cell viability of DLBCL cells decreased progressively with prolonged exposure to CHI and increasing drug concentration. Specifically, the 48 h IC50 values of CHI for RI‐1, SU‐DHL‐8, and OCI‐LY3 cells were 1.75 ± 0.20 μM, 0.26 ± 0.04 μM and 0.98 ± 0.12 μM, respectively (Figure 1A,B). Then, the roles of CHI in the cell cycle and apoptosis induction were analyzed using PI staining and Annexin V/PI double‐staining. After a 48‐h treatment with CHI, nuclear DNA staining revealed a reduction in the proportion of DLBCL cells in the S phase and an arrest in the G1 phase (Figure 1C,D). Apoptosis was significantly induced in RI‐1 and SU‐DHL‐8 cells after the same treatment period, while OCI‐LY3 cells exhibited necrosis rather than apoptosis (Figure 1E,F). In addition, we further determined the expression of some apoptosis‐related proteins by western blotting. The results demonstrated upregulation of the activated form of caspase‐3 protein with increasing concentrations of CHI, indicating its ability to induce caspase‐3 mediated cell apoptosis (Figure 1G,H).

### 3.2 CHI Reduces Mutant p53 Protein Levels in DLBCL Cells

**FIGURE 2:**
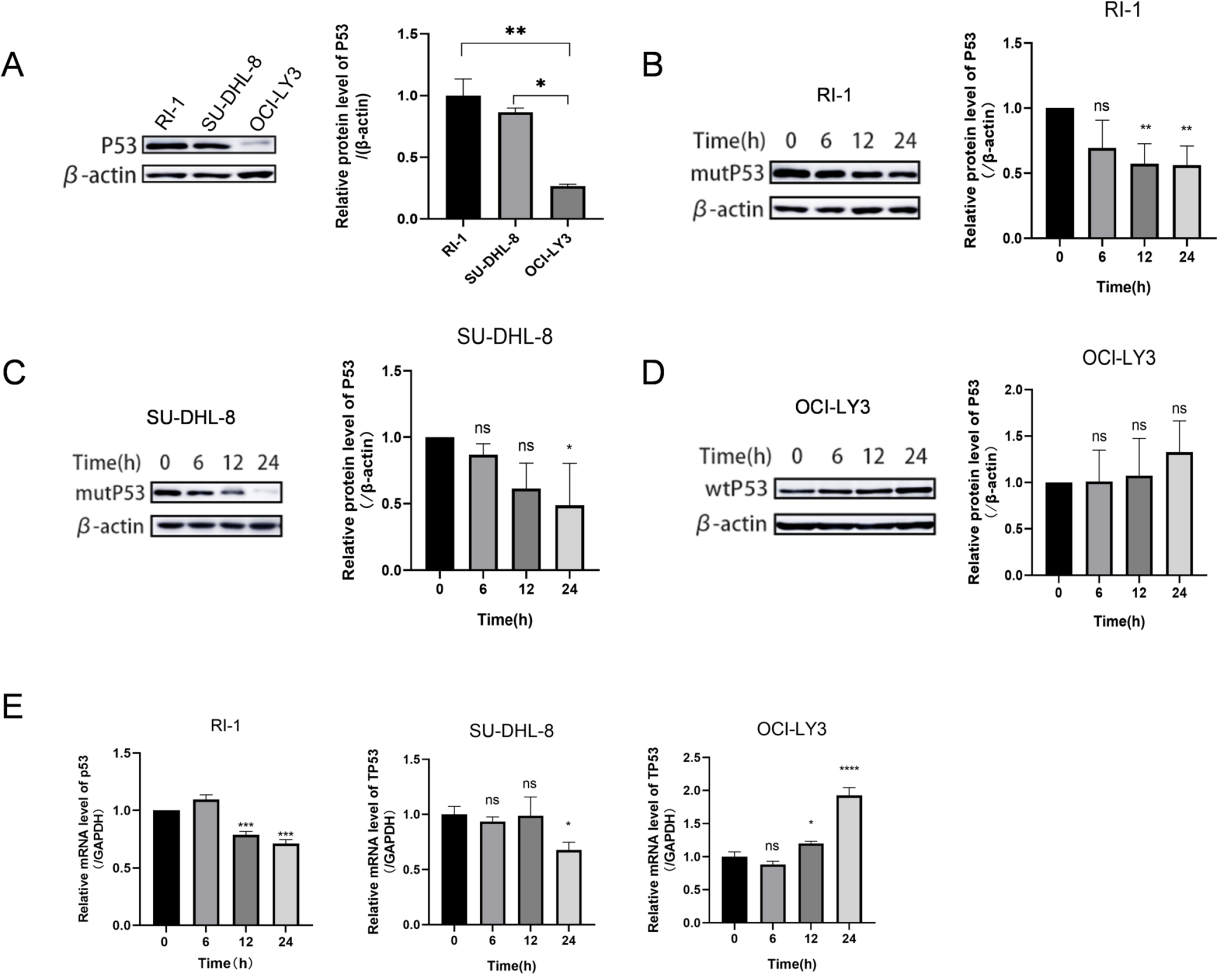
CHI reduces mutant p53 protein levels in DLBCL cells. (A) The expression of p53 in RI‐1, SU‐DHL‐8, and OCI‐LY3 cells. (B‐D) The protein level of p53 in DLBCL cells after 6 h, 12 h, and 24 h was treated with different concentrations of CHI. RI‐1, SU‐DHL‐8, and OCI‐LY3 cells were treated with 2 μM, 0.25 μM, and 1 μM CHI, respectively. Quantitative analysis of the levels of p53 protein. Columns represent the mean from three parallel experiments (mean ± SEM, *n* = 3). ns *p* > 0.05, *p < 0.05, **p < 0.01. (E) The mRNA level of *TP53* in DLBCL cells after 6 h, 12 h, and 24 h was treated with different concentrations of CHI. RI‐1, SU‐DHL‐8, and OCI‐LY3 cells were treated with 2 μM, 0.25 μM, and 1 μM CHI, respectively. Columns represent the mean from three parallel experiments (mean ± SEM, *n* = 3). ns *p* > 0.05, *p < 0.05, ****p < 0.0001.

Studies have shown that the level of p53 protein in normal cells is kept low by being reduced by the E3 ubiquitin ligase MDM2, but mutant p53 protein (mutp53) is expressed at higher levels in tumor cells than wild‐type p53 (wtp53) protein is in normal cells, suggesting that mutp53 is more stable than wtp53 and accumulates in tumor cells [17]. We therefore further examined the effect of CHI on p53 expression in DLBCL cells. After analyzing the basic p53 protein in three DLBCL cell lines, it was observed that the levels of p53 protein in OCI‐LY3 cells with wild‐type p53 were significantly lower than those in RI‐1 and SU‐DHL‐8 cells with p53 mutations (Figure 2A). Following a 24‐h treatment with CHI, the levels of p53 protein in p53 mutant RI‐1 and SU‐DHL‐8 cells were notably reduced, while there was a slight increase in wild‐type OCI‐LY3 cells (Figure 2B–D). This study assessed the mRNA level of the *TP53* gene using RT‐qPCR. After 24 h of CHI administration, a significant decrease in *TP53* mRNA levels was observed in both the SU‐DHL‐8 and RI‐1 cell lines. However, there was a significant increase in the *TP53* mRNA level of OCI‐LY3 cells (Figure 2E), suggesting that CHI could reduce the levels of mutant p53 protein but not wild‐type p53 protein levels.

### 3.3 CHI and DOX Exhibit a Synergistic Effect in Decreasing Cell Viability

**FIGURE 3:**
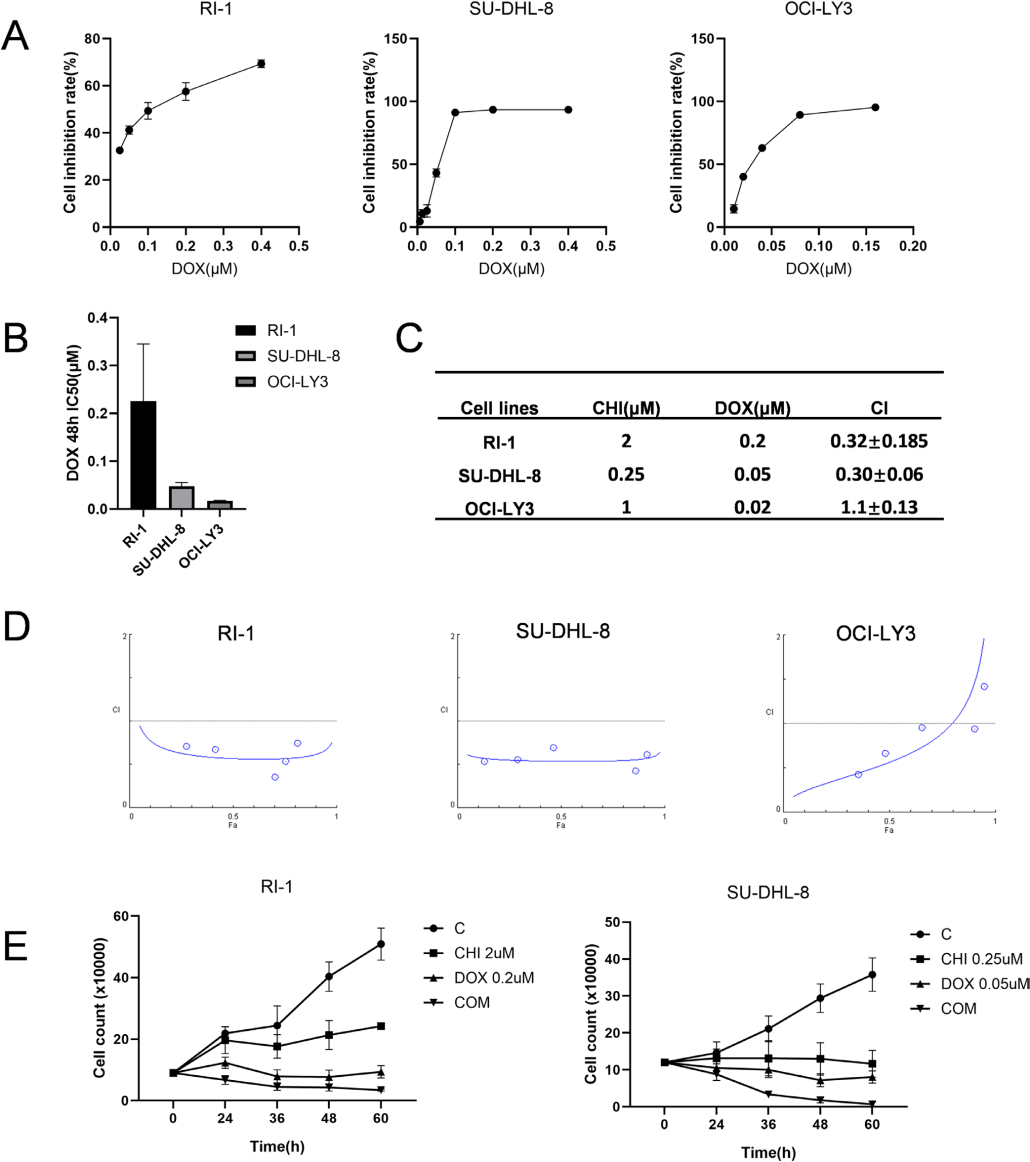
CHI and DOX act synergistically to reduce cell viability. (A) The inhibition rates of DLBCL cells treated with different concentrations of DOX for 48 h. (B) The IC_50_ values were calculated by GraphPad Prism 9.0. Columns represent the mean from three parallel experiments (mean ± SEM, *n* = 3). (C) The combination index of CHI and DOX on DLBCL cells. Data represent the mean ± SEM from three independent experiments. (D) The Fa‐CI plot of DLBCL cells treated with fixed ratio concentrations of CHI and DOX for 48 h. (E) The viable cell density of RI‐1 and SU‐DHL‐8 cells after the use of CHI and DOX. Data represent the mean ± SEM from three independent experiments.

Both CHI and DOX have currently used as clinical drugs. We first explored the inhibitory effect of DOX on the proliferation of DLBCL cells and calculated the IC50 values. The results show that the IC_50_ values of RI‐1, SU‐HDL‐8, and OCI‐LY3 cells treated with DOX for 48 h were 0.23 ± 0.069 μM, 0.048 ± 0.004 μM and 0.017 ± 0.001 μM respectively (Figure 3A,B). Cells were exposed to various concentrations of CHI alone, DOX alone, or CHI plus DOX for 48 h, followed by CCK8 assays to assess cell viability (Figure 3C,D). The CI value was calculated using the IC_50_ values of each drug according to the method of Chou Talalay in order to evaluate the potential synergistic enhancement of cytotoxicity toward RI‐1, SU‐HDL‐8, and OCI‐LY3 cells by the combination of CHI and DOX. The results showed that the CI values of RI‐1 and SU‐DHL‐8 cells were 0.32 ± 0.185 and 0.30 ± 0.06, respectively, indicating a strong synergistic effect, while the CI value of OCI‐LY3 cells was 1.1 ± 0.13, indicating an additive effect. These results show that the combination of chidamide and doxorubicin has a significant synergistic anti‐tumor effect on DLBCL cells with p53 mutation, but only an additive effect on DLBCL cells without p53 mutation.

We then stained RI‐1 and SU‐HDL‐8 cells with trypan blue to assess the number of living cells at 0, 24, 36, 48, and 60 h after treatment with CHI and/or DOX treatment, and converted the results into live cell density. Compared to the control group, cell proliferation was significantly inhibited in the monotherapy group. At all time points, the viable cell density in the combination group was consistently lower than in the single therapy group and decreased over time, indicating that the combination of CHI and DOX significantly inhibited DLBCL cell proliferation and promoted cell death (Figure 3E).

### 3.4 CHI Inhibited the Senescence of DLBCL Cells Induced by DOX

**FIGURE 4:**
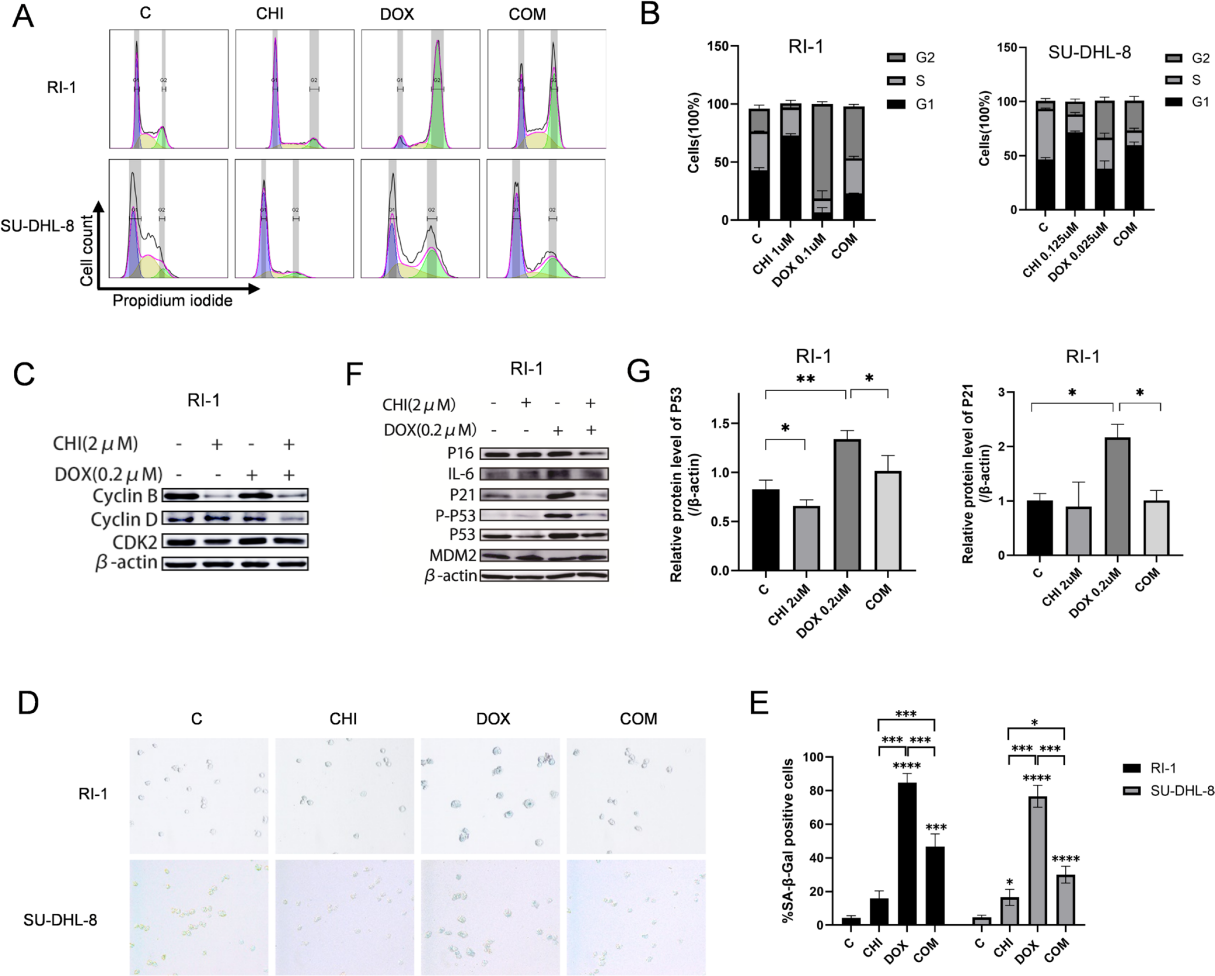
CHI inhibited the senescence of DLBCL cells induced by DOX. (A) RI‐1 and SU‐DHL‐8 cells were treated with CHI and/or DOX for 48 h. DNA was stained by PI and the DNA content was measured by flow cytometry. (B) Quantitative analysis of the proportion of cells in each cycle. Data represent the mean ± SEM from three independent experiments. (C) Protein expression levels of Cyclin B, Cyclin D, and CDK2 in RI‐1 cells were determined by western blot after treatment with drugs for 24 h. (D) SA‐β‐Gal activity was measured by SA‐β‐Gal staining in RI‐1 and SU‐DHL‐8 cells treated with CHI and/or DOX for 48 h. (E) Quantitative analysis of the SA‐β‐Gal activity. Columns represent the mean from three parallel experiments (mean ± SEM). (F) Expression changes of p53, p‐p53, p21, p16, and IL‐6 in RI‐1 cells were performed by western blot. RI‐1 cells were treated with CHI and/or DOX for 48 h. (G) Quantitative analysis of the levels of p53 and p21 protein in RI‐1 cells. Columns represent the mean from three parallel experiments (mean ± SEM, *n* = 3). ns *p* > 0.05, *p < 0.05, **p < 0.01, ***p < 0.001, ****p < 0.0001.

We next investigated the inhibitory effect of CHI combined with DOX on the cell cycle arrest in DLBCL cells. Results showed that both CHI and DOX could induce cell cycle arrest, with CHI blocking cells in the G1 phase and DOX blocking cells in the G2 phase (Figure 4A). Quantitative data showed that compared with the control group, RI‐1 cells showed stronger G2 phase arrest after the combination of the two drugs, while SU‐DHL‐8 cells significantly increased the proportion of cells in G1 and G2 phases and decreased in S phase cells (Figure 4B). Furthermore, we explored the effect of this combination on the expression of proteins critical for G2 phase progression. The treatment with CHI resulted in the downregulation of Cyclin B protein after 24 h, while the expression of Cyclin D remained unaltered. Upon combination treatment with CHI and DOX, both Cyclin B and Cyclin D proteins were downregulated, although there was no significant change in CDK2 levels (Figure 4C).

Then, we continued to investigate the effect of CHI with DOX on the senescence of DLBCL cells. Results showed that after 48 h treatment with DOX, both RI‐1 and SU‐DHL‐8 cells exhibited significant senescence characteristics, including increased cell volume and upregulated SA‐β‐Gal activity with noticeable blue staining. In contrast, CHI treatment for the same duration did not induce significant changes in senescence markers. The combination of both drugs resulted in the absence of blue staining, indicating a lack of cellular senescence (Figures 4D,E and S1A,B). Furthermore, compared to cells treated with DOX alone, the combination of CHI and DOX led to decreased levels of senescence marker proteins p21, p16, and IL‐6 (Figures 4F and S1C,E). Additionally, the protein level of p53, which regulates p21 expression, was also significantly reduced by CHI (Figures 4G and S1D,F).

Additionally, to investigate the role of p53 in DOX‐induced cellular senescence, we knocked down p53 expression in RI‐1 cells via lentiviral transduction and assessed the effect of DOX on senescence in the p53‐knockdown cell line; the results revealed that p53 is involved in DOX‐induced senescence of DLBCL cells (Figure S2).

### 3.5 Combination of CHI and DOX Promoted Apoptosis of DLBCL Cells

**FIGURE 5:**
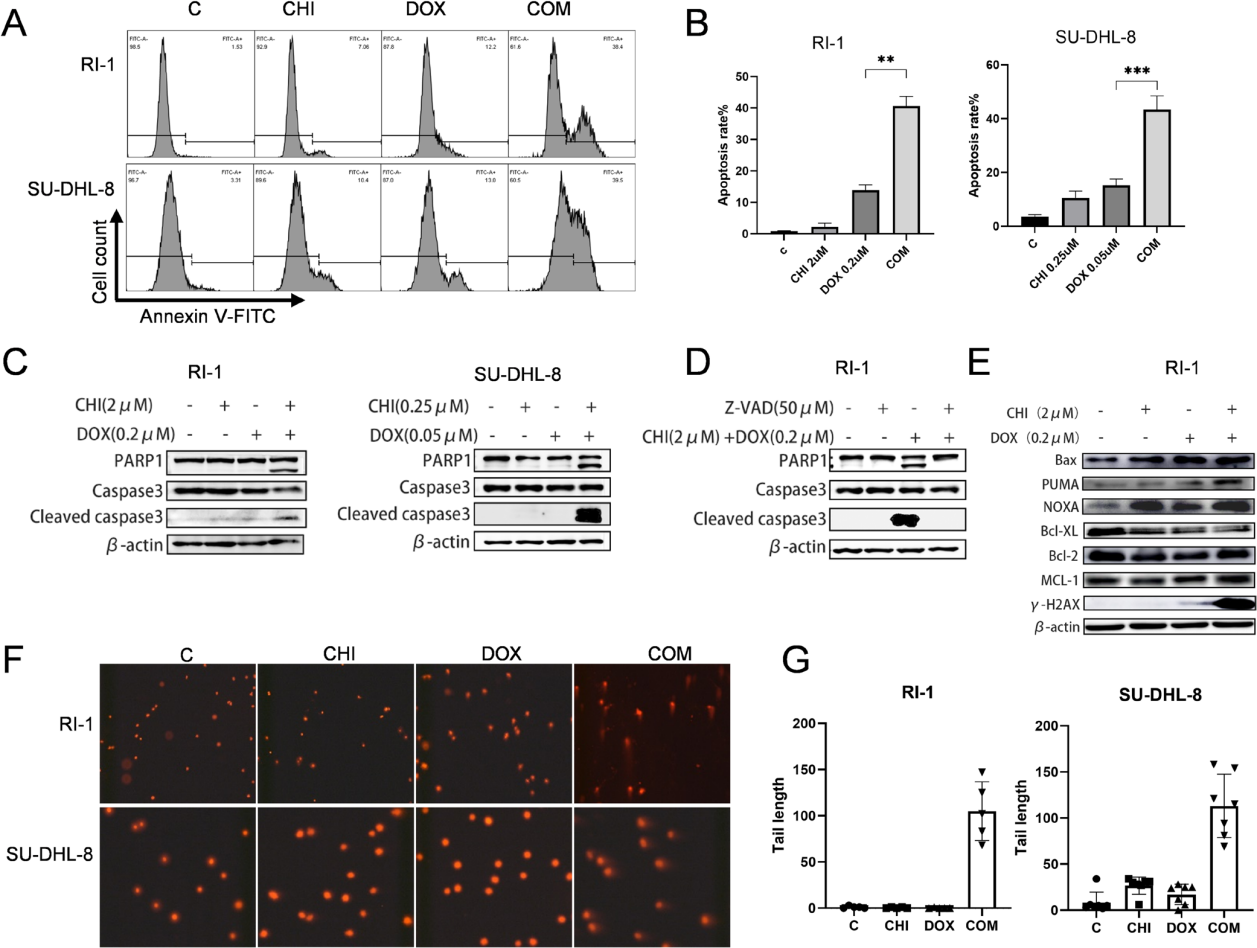
CHI combined with DOX promotes apoptosis of DLBCL cells. (A) Annexin V‐FITC staining of DLBCL cells was determined by flow cytometry. DLBCL cells were treated with CHI and/or DOX for 48 h. (B) Quantitative analysis of apoptosis cells. Data represent the mean ± SEM from three independent experiments. **p < 0.01, ***p < 0.001. (C) DLBCL cells were treated with CHI and/or DOX for 24 h. The expression of PARP‐1 and caspase‐3 were detected by western blot. (D) RI‐1 cells were pretreated with or without 50 μM Z‐VAD‐FMK for 1 h, and then treated with 2 μM CHI and 0.2 μM DOX for 24 h. The expression of PARP‐1 and caspase‐3 were detected by western blot. (E) RI‐1 cells were treated with CHI and/or DOX for 24 h. The expression of Mcl‐1, Bcl‐2, Bcl‐XL, NOXA, PUMA, Bax, and γ‐H2AX were detected by western blot. (F) DNA strand breaks in cells were tested by comet assay treated with CHI and/or DOX for 24 h. (G) Comet tail length of RI‐1 and SU‐DHL‐8 cells was used to measure DNA damage degree.

We then explored the effect of CHI with DOX on the apoptosis of DLBCL cells. The pro‐apoptotic effects of CHI and DOX on RI‐1 and SU‐DHL‐8 cells were assessed using Annexin V staining to monitor apoptosis progression. The combined treatment group demonstrated a significant increase in apoptosis compared to the individual treatments (Figure 5A,B). There was no significant change in apoptotic marker protein levels when the drugs were used individually. However, the combined use resulted in a substantial increase in the protein level of cleaved caspase‐3, leading to downstream cleavage of PARP1 protein and subsequent cell apoptosis (Figure 5C). The caspase inhibitor Z‐VAD‐FMK can reverse the activation of caspase‐3 in RI‐1 cells induced by the combination of CHI and DOX (Figure 5D). Cells treated with CHI plus DOX downregulated the levels of anti‐apoptotic proteins Mcl‐1, Bcl‐2, and Bcl‐XL, while upregulating the levels of pro‐apoptotic proteins NOXA, PUMA, and Bax (Figure 5E). The marker protein for DNA damage, γ‐H2AX, is significantly upregulated in the combination group. Comet assay revealed no comet tailing phenomenon in the monotherapy group, but significant tailing was observed after combination therapy, suggesting that CHI combined with DOX causes substantial DNA damage, but the specific mechanism still needs further exploration (Figure 5F,G).

### 3.6 CHI Plus DOX Exhibits Synergistic Anti‐Tumor Effects and Low Toxicity In Vivo

**FIGURE 6:**
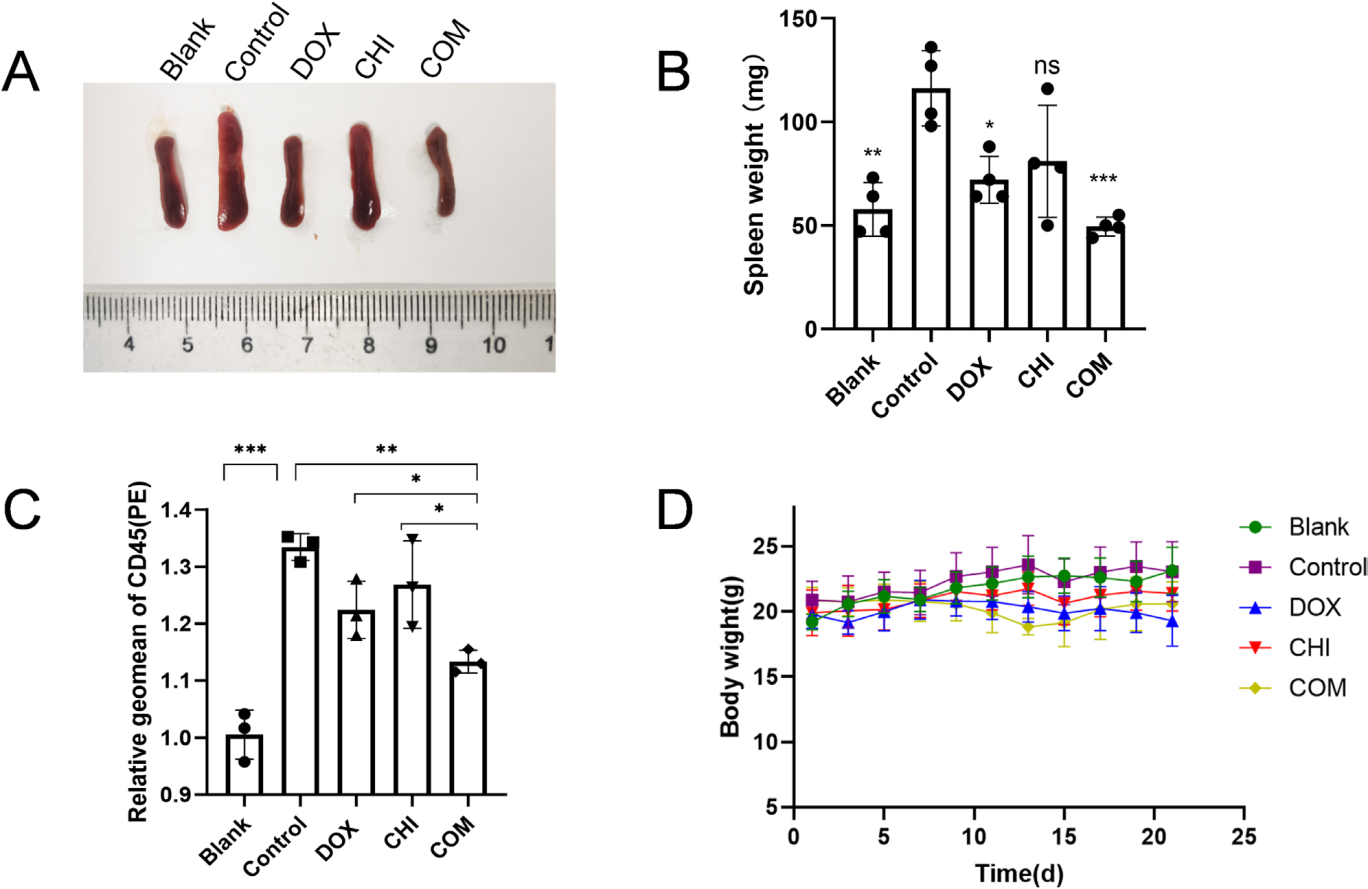
CHI plus DOX exhibits synergistic anti‐tumor effects and low toxicity in vivo. (A) The splenomegaly of SU‐DHL‐8 cell‐bearing nude mice. (B) The spleen weight of SU‐DHL‐8 cell‐bearing nude mice. Data represent mean ± SEM (*n* = 6). *p < 0.05, **p < 0.01, ***p < 0.001. (C) The huCD45 expression of spleen cells from SU‐DHL‐8 cell‐bearing mice in each group was examined by flow cytometry analyses. Data represent the mean ± SEM from three independent experiments. *p < 0.05, **p < 0.01, ***p < 0.001. Significance is determined by one‐way ANOVA analysis. (D) Body weight changes in BALB/c nude mice after intravenous administration of SU‐DHL‐8 cells.

To further explore the synergistic effect of CHI and DOX on inhibiting DLBCL in vivo, this study first established a BALB/c nude mouse hematoma model inoculated with the SU‐DHL‐8 cell line. Mice were divided into 5 groups, namely the blank group, control group, CHI group, DOX group, and combined group. Tumor cells in nude mouse models of hematoma tend to accumulate in the spleen, leading to morphological swelling. After 21 days of administration, the spleens of the nude mice were dissected for observation. It was observed that the spleen volume of the control group inoculated with SU‐DHL‐8 cells was significantly larger than that of the blank group, indicating invasion and subsequent splenomegaly caused by SU‐DHL‐8 cells. The spleens of nude mice in the two monotherapy groups exhibited smaller volumes compared to those in the control group, while the combined group demonstrated the smallest spleen volume and the most effective inhibition of splenic infiltration by lymphoma (Figure 6A,B). CD45 is widely expressed in B cells, and the detection of human CD45 expression in the spleen of nude mice can reflect the growth of SU‐DHL‐8 cells in nude mice. In comparison to the blank group mice that were not inoculated with SU‐DHL‐8 cells, a significant increase in CD45‐positive cells was observed in the spleen of the control group mice, indicating a successful establishment of an animal model for DLBCL cell lymphoma in nude mice. The combination group showed a significant reduction in CD45 cells compared to the control group, with an even more substantial decrease in CD45‐positive cells (Figure 6C). The results indicate a significant synergistic inhibitory effect of the combination of CHI and DOX on DLBCL in vivo. More importantly, the weight changes of nude mice over 21 days after administration demonstrated that the combination of the two drugs had some weight inhibition toxicity, but it was relatively safe and controllable (Figure 6D).

## 4 Discussion

Treatment‐induced senescence combined with senolytics has been a novel strategy in tumor therapy in recent years [18]. Compared to the traditional method of directly killing tumor cells with drugs, this “One‐two punch” strategy is more specific and effective for tumor cells with certain genetic backgrounds [19]. Research has reported that due to defective DNA damage repair caused by p53 mutations, the cell cycle regulator CDC7‐specific inhibitor XL413 can induce senescence in tumor cells carrying p53 mutations. Additionally, XL413 is abnormally sensitive to mTOR inhibitors, and the combination of CDC7 inhibitors and mTOR inhibitors can effectively inhibit the progression of tumors with p53 mutations [20]. Currently, there are few reports on inducing senescence in DLBCL cells and identifying new senolytic drugs. In the present study, we demonstrated that CHI combined with DOX may have a synergistic anti‐DLBCL effect on lysing senescent cells.

CHI is a novel subtype‐selective HDACi [21], and it has been shown that CHI has a combined synergistic effect with the R‐CHOP regimen. CHI can comprehensively inhibit tumor growth by up‐regulating the expression of CD20 and enhancing the pro‐apoptotic effect of R‐CHOP [22]. In this study, we first examined the effects of CHI on DLBCL cells. CHI can inhibit the proliferation of DLBCL cells and induce cell cycle arrest and apoptosis. Previous studies have shown that HDAC inhibitors can inhibit mutant p53 protein levels [23, 24]. The p53 is a tumor suppressor protein, which is a sequence‐specific transcription factor that can bind to determined DNA sequences within the genome and activate the transcription of adjacent genes, regulating the expression of genes related to cell cycle arrest, DNA repair, apoptosis, senescence, and other processes [25]. However, p53 is mutated in most tumor cells, and mutant p53 protein not only loses the wild‐type p53‐dependent tumor suppressor function but also often acquires tumorigenesis‐promoting gain of function (GOF) [26]. Mutations in p53 are frequently observed in DLBCL patients and are associated with a poor prognosis [27]. This study demonstrated that CHI can reduce the levels of mutant p53 protein but has no significant effect on wild‐type p53 protein. A similar phenomenon was observed at the mRNA level, suggesting that CHI may lower p53 levels by enhancing the degradation of mutant p53 protein. By selectively reducing the levels of mutant p53 protein, CHI may have significant implications for DLBCL patients with p53 mutations.

Most first‐line treatments for DLBCL patients include DOX [21]. However, its potential cardiotoxic effects often limit the clinical dosage of DOX. Combination therapy is one way to overcome these limitations [28]. Low‐dose administration of DOX diminishes its cytotoxic effects on tumor cells while enhancing senescence induction [9], thereby increasing the risk of DLBCL recurrence. Consequently, combining drugs capable of lysing senescent cells with DOX for DLBCL treatment may mitigate toxicity and reduce the likelihood of lymphoma relapse, thus holding significant clinical implications [22]. Both CHI and DOX have currently been used as clinical drugs [13, 29]. We found that CHI and DOX exhibit a potent synergistic effect in p53‐mutated DLBCL cells, while no synergistic effects are observed in p53 wild‐type cells. It is speculated that the sensitization of CHI to DOX may be attributed to the decrease in mutant p53 protein levels. In addition, during the regulation of the cell cycle, cyclins activate CDKs to facilitate cell proliferation and division [23]. The study showed that the combination of CHI and DOX led to decreased levels of Cyclin B and Cyclin D proteins but had no significant impact on the protein levels of the cyclins‐activated substrate CDK2, suggesting that the combination can impede cell cycle progression by inhibiting cyclins rather than directly affecting CDK protein levels. When cells undergo prolonged cycle arrest, they enter a state of senescence [30]. This study revealed that low‐dose DOX induced senescence in DLBCL cells. Interestingly, when DOX was combined with CHI, the positive rate of SA‐β‐Gal staining in DLBCL cells decreased significantly, and the expression of senescence‐related proteins p‐p53, p53, p21, p16, and IL‐6 was down‐regulated, indicating that CHI could reverse senescence induced by DOX, but the exact mechanism remains to be further explored.

The p53 protein plays a crucial role in DNA damage repair [31]. Low‐dose DOX alone did not cause DNA damage, possibly due to the upregulation of p53 for DNA repair. The combination of CHI and DOX inhibited the level of p53 protein, thereby hindering its DNA repair function and resulting in DNA damage and apoptosis in DLBCL cells. Moreover, the p21 protein plays a crucial role in determining the pathways of cell senescence or apoptosis; it can induce senescence by inhibiting cell apoptosis [32]. Knocking out p21 in senescent cells activates caspase through a cascade reaction, leading to programmed cell death [33]. Additionally, a study revealed that low concentrations of DOX upregulate the expression of p21 after p53 activation, resulting in cellular senescence, while high concentrations of DOX directly activate p53 and induce cell apoptosis while suppressing the expression of p21 [34]. We found that CHI inhibits DOX‐induced cell senescence by reducing the level of mutant p53 protein and preventing its transcriptional activation of p21. The combination of CHI and DOX significantly decreased the level of p21 protein, potentially causing DNA damage in DLBCL cells induced by low‐dose DOX to bypass the senescent state and enter directly into apoptotic processes. The results show that the combination of CHI and DOX significantly induced apoptosis in DLBCL cells and was reversible by the caspase inhibitor Z‐VAD‐FMK. Endogenous apoptosis is regulated by the distribution and interaction of pro‐apoptotic and anti‐apoptotic Bcl‐2 proteins, which can lead to apoptosis through upregulation of pro‐apoptotic proteins or downregulation of anti‐apoptotic proteins [35]. CHI plus DOX exerts a pro‐apoptotic effect by upregulating the levels of pro‐apoptotic proteins NOXA, PUMA, and Bax, while downregulating the levels of anti‐apoptotic proteins Mcl‐1, Bcl‐2, and Bcl‐XL. This finding is consistent with the overall increase in apoptosis observed in our study. The types of cell lines used in this study are limited and may require further exploration in more DLBCL cell lines or even patient‐derived models. Furthermore, even in the combination group of the two drugs, the mice's body weight increased steadily, suggesting that the combined treatment of CHI and DOX was well tolerated.

Taken together, our findings demonstrate that the combination of CHI and DOX exhibits significant synergistic antitumor effects in vitro, highlighting its potential as an effective and precise preclinical therapeutic strategy for the treatment of DLBCL. These results warrant further clinical investigation. However, the in vivo studies reveal a more modest impact, as evidenced by the reduction in spleen size. This indicates that while the combination therapy shows promise in cell culture, its efficacy in the in vivo setting may be influenced by additional biological complexities. Future research will focus on elucidating these factors and optimizing the therapeutic approach to enhance its clinical relevance.

## Author Contributions

Y.Y., Y.L., and Y.L. performed research, analyzed data, and wrote the paper; M.Z. performed research and analyzed data; H.C. and Z.Z. performed research; Z.T. collected data and performed statistical analysis; H.H. provided guidance on the project design; J.X. provided the blood samples; and H.L. conceptualized the project and directed the experimental design and data analysis.

## Ethics Statement

The animal use protocol has been reviewed and approved by the Institutional Animal Care and Use Committee (IACUC), Approval No. 1ACUC‐2103045.

## Conflicts of Interest

The authors declare no conflicts of interest.

## Supporting information


**Figure S1:** CHI inhibited the senescence of DLBCL cells induced by DOX.

**Figure S2:** p53 is involved in DOX‐induced senescence of DLBCL cells.

## Data Availability

The authors have nothing to report.
