# Supplementary figures and images for: Chidamide Accelerates the Death of Senescence‐Like Diffuse Large B‐Cell Lymphoma Cells With TP53 Mutation Induced by Doxorubicin
Source: FASEB J. 2025 Oct 22;39(20):e71167. doi: 10.1096/fj.202500962RR (PMC12541562; doi:10.1096/fj.202500962RR)

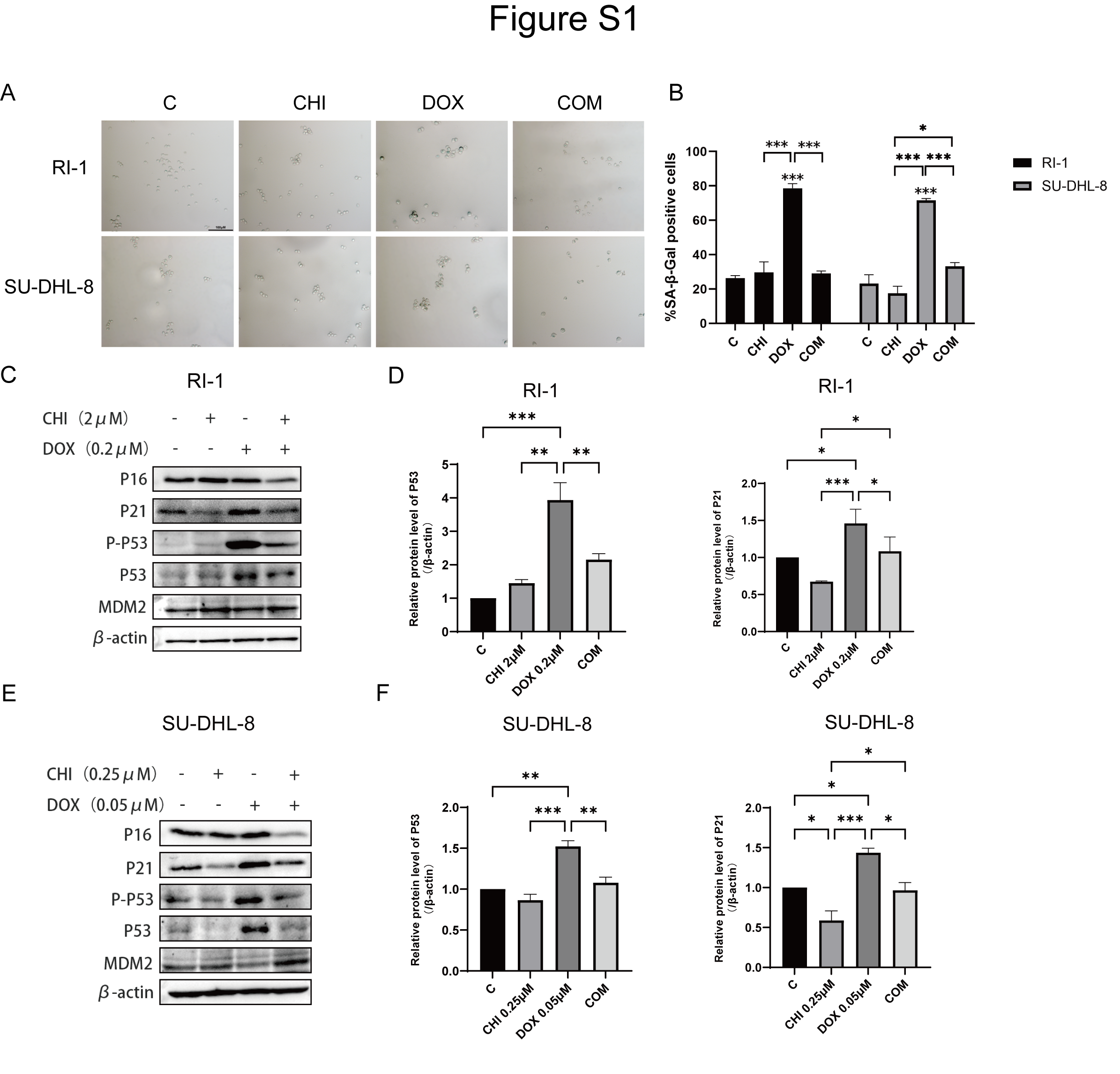

Supplement: Supplementary file 1 — Figure S1: CHI inhibited the senescence of DLBCL cells induced by DOX. [file FSB2-39-e71167-s001.tif]

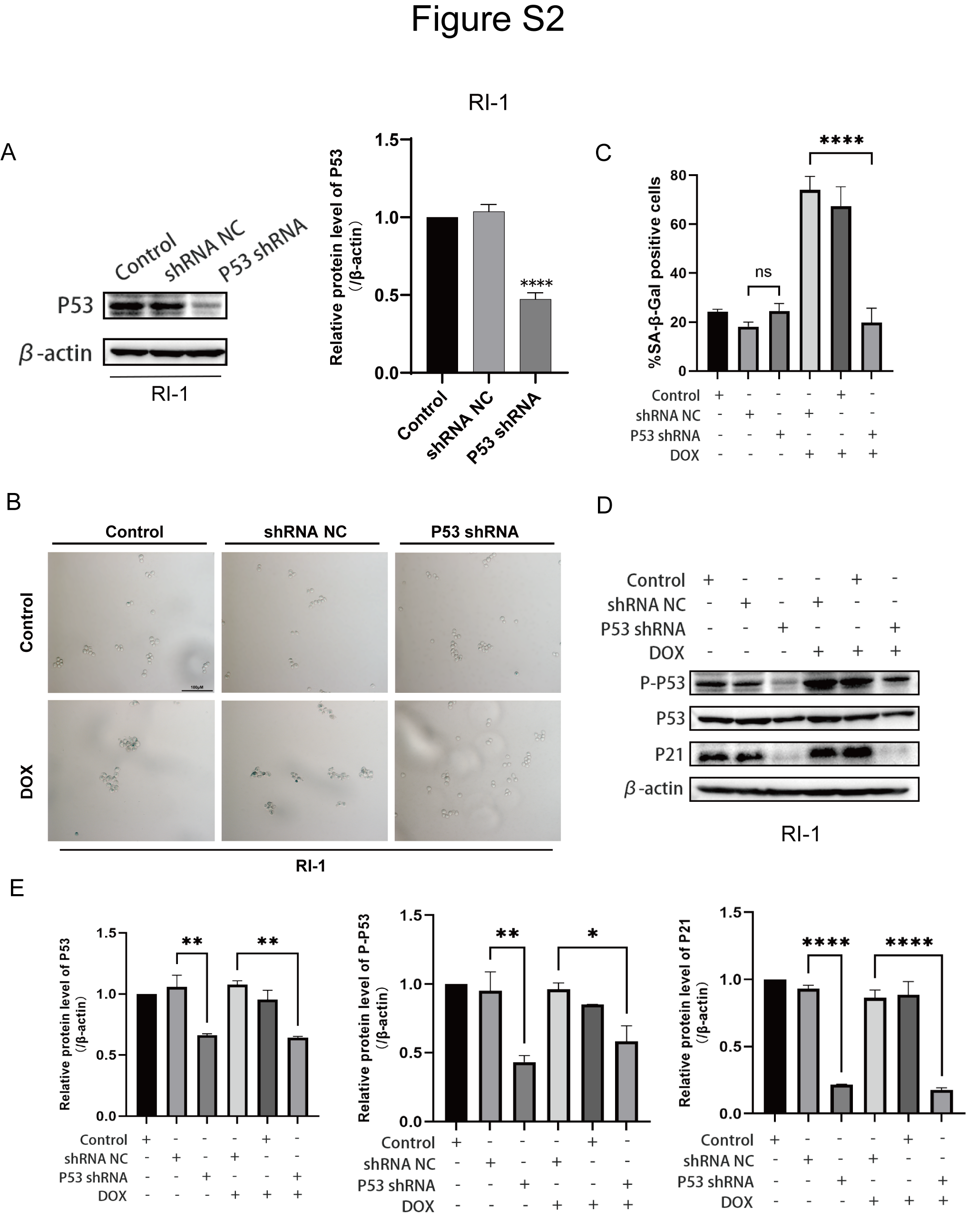

Supplement: Supplementary file 2 — Figure S2: p53 is involved in DOX‐induced senescence of DLBCL cells. [file FSB2-39-e71167-s002.tif]
